# Protective Effects of Hydroxychloroquine against Accelerated Atherosclerosis in Systemic Lupus Erythematosus

**DOI:** 10.1155/2018/3424136

**Published:** 2018-02-18

**Authors:** Alberto Floris, Matteo Piga, Arduino Aleksander Mangoni, Alessandra Bortoluzzi, Gian Luca Erre, Alberto Cauli

**Affiliations:** ^1^Rheumatology Unit, University Clinic and AOU of Cagliari, Monserrato, Italy; ^2^Department of Clinical Pharmacology, College of Medicine and Public Health, Flinders University and Flinders Medical Centre, Adelaide, Australia; ^3^Department of Medical Sciences, Section of Rheumatology, University of Ferrara and Azienda Ospedaliero-Universitaria Sant'Anna di Cona, Ferrara, Italy; ^4^Rheumatology Unit, Department of Clinical and Experimental Medicine, University Hospital (AOUSS) and University of Sassari, Sassari, Italy

## Abstract

Cardiovascular (CV) morbidity and mortality are a challenge in management of patients with systemic lupus erythematosus (SLE). Higher risk of CV disease in SLE patients is mostly related to accelerated atherosclerosis. Nevertheless, high prevalence of traditional cardiovascular risk factors in SLE patients does not fully explain the increased CV risk. Despite the pathological bases of accelerated atherosclerosis are not fully understood, it is thought that this process is driven by the complex interplay between SLE and atherosclerosis pathogenesis. Hydroxychloroquine (HCQ) is a cornerstone in treatment of SLE patients and has been thought to exert a broad spectrum of beneficial effects on disease activity, prevention of damage accrual, and mortality. Furthermore, HCQ is thought to protect against accelerated atherosclerosis targeting toll-like receptor signaling, cytokine production, T-cell and monocyte activation, oxidative stress, and endothelial dysfunction. HCQ was also described to have beneficial effects on traditional CV risk factors, such as dyslipidemia and diabetes. In conclusion, despite lacking randomized controlled trials unambiguously proving the protection of HCQ against accelerated atherosclerosis and incidence of CV events in SLE patients, evidence analyzed in this review is in favor of its beneficial effect.

## 1. Introduction

Systemic lupus erythematosus (SLE) is a chronic autoimmune inflammatory disease characterized by a broad range of clinic manifestations and serologic findings [1, 2]. The prevalence of SLE ranges between 28.3 and 149.5 cases per 100,000 people and is higher in females of childbearing age [3]. Patients with SLE have a 2 to 3 times increased risk of premature death. Cardiovascular disease (CVD) is the leading cause of mortality regardless of time after diagnosis [4, 5]. The overall risk of myocardial infarction (MI) in SLE patients is 10-fold higher than that in the general population; however, it is much greater in young SLE women aged 35–44 years old, who are over 50 times more likely to have a MI, than in age-matched women without SLE [6, 7]. Noteworthy, the increased awareness of the burden of CVD in patients with SLE has not yet translated into decreased rates of hospitalization for acute MI or stroke [8, 9].

The higher risk of CVD in SLE patients is mostly related to accelerated atherosclerosis, which leads to clinical symptoms and manifestations at an earlier age compared to the general population [10]. Despite the pathobiological bases of accelerated atherosclerosis are not fully understood, it is thought that this process is driven by the complex interplay between autoimmunity, inflammation, vascular repair, traditional risk factors, and therapeutic agents [10, 11]. As a result, not surprisingly, the traditional Framingham cardiac risk factors do not fully explain the increased prevalence of CVD observed in SLE [6, 12–14]. Moreover, multiple SLE-related features of autoimmunity have been associated with accelerated atherosclerosis [10, 11, 15, 16].

Hydroxychloroquine (HCQ) has been used for more than 50 years in the treatment of SLE patients. Over the last decades, an increasing number of *in vitro* and *in vivo* studies have highlighted the potential protective effect of HCQ against CVD through multiple mechanisms of action. This review discusses the role of SLE-related and SLE-unrelated factors in the pathophysiology of accelerated atherosclerosis, the pharmacology of HCQ, and the available evidence regarding the effects of this agent in reducing CV risk in SLE patients.

## 2. SLE and Accelerated Atherosclerosis

Roman et al. reported an increased prevalence of atherosclerosis, as determined by ultrasound assessment of carotid plaques, in patients with SLE (RR 2.4; 95% confidence interval (CI), 1.7–3.6; *P* < 0.001), particularly in those younger than 40 years which prevalence was 5.6 times higher than healthy controls [17]. Similarly, Asanuma et al. found a significantly higher prevalence of coronary calcification (OR 9.8, 95%CI 2.5–39.0, *P* = 0.001) and greater coronary artery calcium scores (*P* < 0.001) in SLE patients than in healthy controls [18].

Longer disease duration (OR 2.14, 95%CI 1.28–3.57; *P* = 0.004) and higher disease-related Systemic Lupus International Collaborating Clinics (SLICC)/damage index (SDI) (OR 1.26 per SDI point score, 95%CI 1.03–1.55, *P* = 0.03) were identified as independent predictors of carotid plaque in SLE [17]. In some studies, lupus disease activity was significantly associated with subclinical measures of atherosclerosis in univariate analysis, but its independent effect was not confirmed in multivariate analysis [19–21].

## 3. Interplay between SLE and Atherogenesis

The increasing evidence that both adaptive and innate immunity take part in the initiation and progression of atherosclerosis suggests that the dysregulation of the immune system of SLE could play an independent role in atherogenesis (Table 1) [22].

### 3.1. Endothelial Dysfunction

Endothelial dysfunction is one of the earliest signs of atherosclerosis [16, 23], resulting in increased expression of adhesion molecules and impaired vasodilation [24]. A recent meta-analysis, of 25 case-control studies involving 1313 SLE patients and 1012 healthy controls, confirmed that patients with SLE who are naïve of cardiovascular disease have impaired endothelial function as determined by brachial artery flow-mediated dilation [25].

An imbalance between circulating apoptotic endothelial cells (ECs), indicative of vascular damage, endothelial progenitor cells (EPCs), and circulating myelomonocytic angiogenic cells (CACs), expression of vascular repair mechanisms, was described in SLE patients [26, 27]. Such findings correlate with the presence of endothelial dysfunction (beta = −4.5, *P* < .001) assessed by brachial artery flow-mediated dilation [26].

Both endothelial damage and the initiation of the atherogenic process are influenced by the redox environment. Patients with SLE have increased concentrations of reactive oxygen species (ROS) and decreased antioxidant defense mechanisms which provide a favorable environment for oxidation of lipoproteins and atherosclerosis development [28, 29]. Moreover, a positive correlation between SLE disease activity and oxidative stress was observed in some studies [28, 30, 31], but not in others [32, 33].

Further potential mechanisms involved in endothelial dysfunction in SLE include alterations in lipid profile with increased oxidized LDL (ox-LDL) and proinflammatory high-density lipoproteins (HDL) [11], high frequency of low-density granulocytes (LDG) with direct toxic effect on the endothelium [34], renal involvement, and antiphospholipid antibodies [35, 36].

### 3.2. Monocytes and T-Cell Recruitment and Activation

Due to the overexpression of adhesion molecules and the increased chemokine releasing by activated ECs, monocytes can migrate into the intima and differentiate into macrophages. The uptake of ox-LDL by scavenger receptors leads to a further transformation into foam cells that secrete proinflammatory cytokines under the toll-like receptor (TLR) stimuli [22]. Macrophage activation, as assessed by serum neopterin measurement, was demonstrated to be increased in SLE patients (median (IQR) serum neopterin nmol/L: 8.0 (6.5–9.8) versus 5.7 (4.8–7.1) in SLE and healthy controls, resp.) [37] and to correlate with SLE disease activity [38, 39]. However, a significant association with coronary calcium in SLE patients was not observed [37].

T-cells, consisting predominately of CD4+ T helper 1, are recruited to nascent atherosclerotic plaques similarly to monocytes and represent approximately 7–17% of the cells in the lesion [40]. T-cells have been shown to be hyperactive in lupus patients, with reduced apoptosis rate and increased survival [41–43]. In support of the role of CD4+ T-cells in the link between SLE and atherosclerosis, Stanic et al. demonstrated an increased infiltration of CD4+ T-cells into the atherosclerotic lesions of LDLr^−/−^ mice following transfer of bone marrow from lupus-susceptible mice [44].

### 3.3. Toll-Like Receptors

The toll-like receptors (TLRs), a class of pattern recognition receptors expressed on multiple cells involved in innate immunity, were demonstrated to be involved in atherogenesis [45, 46]. Edfeldt et al. found that the expression of TLR1, TLR2, and TLR4 was markedly enhanced in human atherosclerotic plaques [47]. Miller et al., in their *in vitro* experiments, reported that the binding of TLR4 and CD14 to ox-LDL on macrophages inhibits the phagocytosis of apoptotic cells, upregulates the expression of the scavenger receptor, and increases the uptake of ox-LDL [48].

Recent studies described a dysregulated activation of TLR2 and TLR4 in SLE patients, resulting in upregulated production of autoantibodies and cytokines [49]. Moreover, the endogenous anti-DNA antibody immune complexes typical of SLE can bind TLR7 and TLR9 on active plasmacytoid dendritic cells (DCs) and promote the release of IFN*α*. This leads to the recruitment of activated inflammatory cells, self-perpetuating the process of inflammation and plaque formation [46].

### 3.4. Cytokines

Many cytokines are involved both in atherosclerosis and SLE pathogenesis. IFN*α* is a multifunctional cytokine which plays a pivotal role in SLE pathogenesis. IFN*α* concentrations are increased in SLE patients, associate with disease activity [50], and seem to be involved in endothelial dysfunction. Denny et al. demonstrated that IFN*α* induces EPC and CAC apoptosis and skews myeloid cells toward nonangiogenic phenotypes, whilst neutralization of IFN pathways led to a normalization of the EPC/CAC phenotype [27, 43]. Recently, IFN*α* has been claimed to serve as a proatherogenic mediator through repression of endothelial NO synthase-dependent pathways promoting the development of endothelial dysfunction and cardiovascular disease in SLE [51].

IFN*γ*, a key regulator of immune function, was demonstrated to be highly expressed and to play a crucial role both in SLE and in atherosclerosis [52, 53]. IFN*γ* participates in atherogenesis by stimulating ECs and macrophage activation, proinflammatory mediator production, and adhesion-molecule expression and by inhibiting smooth muscle cell proliferation and collagen production [22, 54].

Other cytokines overexpressed in SLE, such as TNF-*α*, IL-17, and IL-6, participate in the initiation and perpetuation of the atherosclerotic process by stimulating the activation of macrophages, inducing the secretion of matrix metalloproteinases, upregulating the expression of adhesion molecules on the ECs, increasing the concentration of chemotactic messengers, and affecting the proliferation of smooth muscle cells [15, 55–59]. In SLE, serum TNF-*α* concentrations have been reported to be elevated and to correlate with CVD and altered lipid profiles [60, 61].

### 3.5. Reduced Protective Effect of High-Density Lipoproteins

HDL have atheroprotective effects through the inhibition of oxidative modification of LDL, stimulation of reverse cholesterol transport, and attenuation of endothelial dysfunction. During the acute phase of inflammation, HDL can be converted from anti-inflammatory to proinflammatory molecules that promote LDL oxidation [62, 63]. McMahon et al. found that a higher proportion of SLE patients had proinflammatory HDL (44.7% of SLE patients versus 4.1% of controls, *P* < 0.006 between all groups), which correlated with ox-LDL concentrations (*r* = 0.37, *P* < 0.001) and coronary artery disease (*P* < 0.001) [64].

The prevalence of antibodies against apolipoprotein A1 (anti-ApoA-1), the main component of HDL, is significantly higher in patients with acute coronary syndrome (21%) and in patients with SLE and/or antiphospholipid syndrome (13–32%), than in healthy subjects (1%) [65, 66]. Although the direct demonstration of a cause-effect relationship is needed, the high prevalence of anti-ApoA-1 autoantibodies in SLE patients is supposed to play a role in accelerated atherosclerosis.

## 4. Increased Prevalence of Traditional Cardiovascular Risk Factors in SLE

Some of the traditional risk factors for atherosclerosis, such as dyslipidemia, diabetes, and hypertension, have an increased prevalence in SLE patients [67].

### 4.1. Dyslipidemia

SLE patients exhibit an increased incidence of proatherogenic lipid profile, consisting in low concentrations of HDL and high concentrations of triglycerides, total cholesterol, and LDL [43]. The increased prevalence of dyslipidemia in SLE may be due to both steroid therapy and disease-related pathogenetic mechanisms, including increased C-reactive protein levels, cytokine release (e.g., TNF-alpha and IL-6), and antibodies against lipoprotein lipase (LPL) affecting the balance between pro- and antiatherogenic lipoproteins [68]. In 918 SLE patients of the Systemic Lupus International Collaborating Clinics' cohort, the prevalence of hypercholesterolemia was 36% at diagnosis and 60% 3 years later [69]. Moreover, in the same cohort, hypercholesterolemia was significantly associated with CV events (OR = 4.4, 95%CI 1.51–13.99) [70].

### 4.2. Hypertension

Hypertension is an independent risk factor CV in SLE (OR 5.0; 95%CI 1.3–18.2) [70]. In a case-control study, Bruce et al. reported a 2.59 RR (95%CI 1.79–3.75) of hypertension in women with SLE [12]. In a multivariate analysis, Doria et al. found that hypertension was associated with atherosclerosis by means of higher carotid intima-media thickness in SLE patients [21].

### 4.3. Diabetes and Insulin Resistance

An increased prevalence of insulin resistance and diabetes was reported in several studies [70–72], but not in all [73]. Bruce et al. reported a 6.6 RR (95%CI 1.36–26.53) of diabetes, which is an established risk factor for CVD, in SLE women [12].

An unbalance in adipokine production, consisting of lower concentrations of adiponectin and higher concentrations of leptin, was proposed as a potential cause of the increased prevalence of insulin resistance in SLE, as well as corticosteroid use [74]. However, neither insulin resistance nor diabetes has been shown to independently predict CV events in SLE cohorts [70, 72].

Dyslipidemia, hypertension, and insulin resistance can be part of metabolic syndrome that was observed to be more frequent in SLE patients compared with controls (32.4% versus 10.9%; *P* < 0.001) and associated to an increased risk of atherosclerosis by means of aortic pulse wave velocity [75, 76].

## 5. Hydroxychloroquine Pharmacology

HCQ is an antimalarial agent that has been used for many years in treating inflammatory rheumatic diseases, especially SLE and rheumatoid arthritis. HCQ is administered orally as the sulphate salt and, being a weakly basic drug, is rapidly absorbed in the upper gastrointestinal tract with a large volume of distribution. HCQ is then dealkylated by cytochrome P450 enzymes into its active metabolite desethyl-HCQ [77]. The systemic clearance is by renal excretion with a long tissue half-life of 40–50 days. HCQ may take up to 4–6 weeks for the onset of therapeutic action and 3–6 months to achieve the maximal clinical efficacy. The recommended dose of HCQ is 200–400 mg daily or about 5 mg/kg/day in a weight-based regimen [77]. According to Durcan et al. [78], HCQ dosing based on actual body weight, instead of ideal weight, is appropriate for patients with SLE. Blood HCQ concentrations can be measured with available commercial kits, which may help in adherence monitoring and the identification of individualized therapeutic regimens [79].

**Figure 1:**
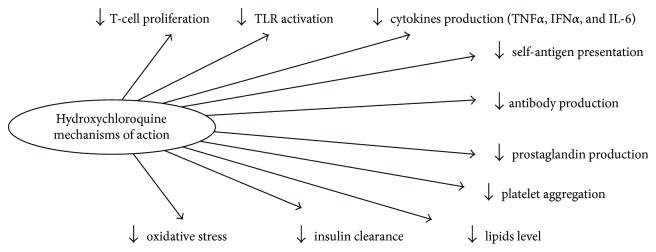
HCQ mechanisms of action.

HCQ has numerous and complex mechanisms of action (Figure 1). The increasing pH in the intracellular compartments (“lysosomotropic action”) favors HCQ-mediated interference with phagocytosis, receptor recycling, antibody production, and selective presentation of self-antigens [67]. Moreover, HCQ blocks T-cell and monocyte proliferation, inhibits TLR signaling, and downregulates cytokine production including TNF-alpha, IL-17, IL-6, IFN*α*, and IFN*γ* [77].

## 6. Hydroxychloroquine Clinical Benefits in SLE

### 6.1. Disease Activity

The first study on HCQ clinical efficacy in SLE randomized 25 patients to continue HCQ on stable dose therapy and 22 patients to switch to placebo for 24 weeks. A lower rate of flare (36% versus 73%, *P* = 0.02; RR 2.5 95%CI 1.1–5.6) was observed in the HCQ group [80]. More recently, Ruiz-Irastorza et al. systematically reviewed the effect of HCQ on lupus activity and identified 8 studies, of which 3 were randomized controlled trials [81]. All studies were of high quality and consistently found lupus disease activity and flares to be significantly reduced in patients treated with HCQ [81, 82].

### 6.2. Atherosclerosis

Some studies did not find any effect of current [20, 83] or past [84–87] treatment with HCQ on the presence of atherosclerosis. On the other hand, Roman et al., in multivariate analysis, found a borderline-independent effect of current or former treatment with HCQ (adjusted OR 0.49; 95%CI 0.21–1.12; *P* = 0.09) in reducing plaque burden, on carotid ultrasound, of SLE patients [17]. Moreover, the current use of HCQ was associated with significantly lower (partial R2 0.025; *P* = 0.032) aortic stiffness, measured by pulse wave velocity, in premenopausal SLE women [88]. Noteworthy, the only study specifically designed to analyze the effect of treatment with HCQ on atherosclerosis, albeit conducted in a relatively small population (*n* = 41), found increased large artery elasticity (13.7 versus 8.3 mmHg × ml × 10; *P* = 0.006) and reduced systemic vascular resistance (14.4 versus 18.4 dyne × sec × 10^−3^; *P* = 0.05) among patients treated with HCQ compared with those receiving corticosteroids only [89]. Overall, the available evidence is inconclusive, mainly as a result of poor study quality and design [81].

### 6.3. Irreversible Target Organ Damage and Survival

The beneficial effects of HCQ on target organ damage and survival in SLE patients have been demonstrated by several high-quality evidence studies [81, 90–93]. For example, HCQ was protective (HR 0.73; 95%CI 0.52 to 1.00) against damage accrual, calculated using the SLICC damage index, in the prospective LUMINA (Lupus in Minorities: nature versus nurture) study cohort, particularly in those patients without damage at baseline (HR 0.55, 95%CI 0.34 to 0.87) (94). In the same cohort, 17% of patients not taking HCQ died during the follow-up versus 5% of those treated with HCQ (*P* < 0.001), accounting for a 0.28 unadjusted OR (95%CI 0.05 to 0.30) and 0.32 adjusted OR (95%CI 0.12 to 0.86) [94]. Moreover, HCQ use was associated with less cerebrovascular damage on brain MRI of SLE patients (OR 0.08; 95%CI 0.01–0.73) [95], less thrombosis (OR 0.31, 95%CI 0.13–0.71) [96], less CV events (HR 0.04, 95%CI 0.004–0.48) [97], and less, albeit not statistically significant, cardiovascular mortality (0% versus 36.8%) [98].

In a multinational Latin American inception cohort, a lower mortality rate was observed in antimalarial users compared with nonusers (4.4% versus 11.5%; *P* < 0.001), and, after adjustment for potential confounders in a Cox regression model, antimalarial use was associated with a 38% reduction in the mortality rate (hazard ratio 0.62, 95%CI 0.39–0.99) [99].

It remains to be established whether HCQ exerts its protective effects on damage accrual and survival in SLE patients through lowering disease activity, preventing atherosclerosis, or both.

## 7. Hydroxychloroquine and SLE-Related Risk Factors for Atherosclerosis

### 7.1. Endothelial Dysfunction

Endothelial dysfunction (ED) is a potentially reversible alteration thus representing an attractive target for CVD prevention and treatment. Gómez-Guzmán et al. [100] found that short-term treatment with HCQ in advanced disease stages is able to reverse large artery ED in a murine model of SLE. This effect was mediated by a reduction of nicotinamide adenine dinucleotide phosphate (NAD(P)H) oxidase activity, which is a major ROS source. Recently, Virdis et al. confirmed that early treatment with HCQ exerts protective effect by decreasing vascular oxidative stress and improving endothelium-dependent relaxation, essentially by preserving the NO-mediated component [101].

### 7.2. Toll-Like Receptor Signaling and Cytokine Production

Evidence that HCQ acts by blocking the nucleic acid-sensing TLRs (TLR3, TLR7, TLR8, and TLR9) is the most important advance in our understanding of its mechanism of action. Nucleic-sensing TLRs, located in intracellular compartments, are activated when interacting with foreign nuclear material presented by specialized molecules such as FC-gamma receptor on DCs or B-cell receptor on the surface of B-cells. HCQ interferes with the TLR7 and TLR9 signaling pathways, reducing the production of IFN*α*, IL-6, and TNF-*α* [102]. It has been postulated that, by altering the lysosomal pH, HCQ prevents TLR functional transformation and activation [103]. However, it is also possible that, by binding nucleic acids, HCQ masks their TLR-binding epitope preventing TLR activation [104].

Beyond the inhibition of TLR signaling, experimental evidence showed that HCQ reduces the concentration of proatherogenic cytokines, such as IFN*α*, IL6, TNF-*α*, IL17, and IL22, in SLE patients through different mechanisms [105, 106]. The observation that HCQ reduces the expression of miR155 in NZB/NZW mice, a SLE animal model, suggests additional therapeutic effects through an epigenetic control of cytokine gene expression [107].

### 7.3. Actions on Immune System Cells and Autoantibody Production

T-cell and B-cell activities may be directly or indirectly affected by HCQ [103]. The HCQ “lysosomotropic action” is responsible for altering the process of self-antigen presentation, whilst preserving that of exogenous antigens, and may also inhibit the intracellular calcium signals after T-cell-receptor stimulation, preventing T-cell activation and proliferation [103, 108]. Furthermore, the inhibition of IFN*α*, IL6, IL17, and TNF-*α* production affects B-cell activation and autoantibody production and favors the differentiation of endothelial cells [103].

The reported HCQ-mediated effects may theoretically reduce the initiation and progression of atherosclerosis by inhibiting the monocyte adhesion to endothelial cells, reducing smooth cell proliferation and favoring vascular repair. However, to date, no study has investigated whether the described effects of HCQ may have a direct benefit in preventing atherosclerosis in SLE patients. More research is warranted to confirm, or refute, this hypothesis.

## 8. Hydroxychloroquine and Traditional Atherosclerosis Risk Factor

### 8.1. Effects on Lipid Profile

The beneficial effect of HCQ on dyslipidemia in patients with SLE has been known for some time. Potential mechanism underlying the beneficial effect of antimalarials on dyslipidemia may be represented by upregulation of LDL receptors with an enhancement of the plasma removal of this lipoprotein [109]. This potential effect of antimalarials would minimize the increased lipoprotein hepatic synthesis induced by steroids [110]. Petri et al. [111] found that HCQ treatment was independently associated with lower serum cholesterol concentrations in multivariate analysis (effect on mg% −8.94; *P* = 0.009). In a cohort of 815 patients, Rahman et al. [13] showed that the lipid lowering effect of antimalarials (mainly HCQ) was higher in patients on a stable dose of steroids and consisted of a reduction in total cholesterol concentrations of 11.3% at 3 months (*P* = 0.0002) and 9.4% at 6 months (*P* = 0.004). Contrasting results have been reported on the different lipoprotein profiles [112–114]. However, two recent prospective studies specifically designed to analyze the effect of HCQ on lipoprotein concentrations, after correction for the confounding effect of other variables, found lower LDL (*P* = 0.036) [113], VLDL (*P* = 0.002), and triglyceride concentrations (*P* = 0.043) and higher HDL concentrations (*P* = 0.03) [114] in patients treated with HCQ.

### 8.2. Effects on Glucose Level

Hypoglycemia has been reported in patients treated with antimalarials. *In vitro* and animal studies, antimalarials affected insulin metabolism, increasing insulin binding to its receptor, altering hepatic insulin metabolism, potentiating insulin action, and reducing the insulin clearance [115–117]. A small randomized study in decompensated diabetic patients showed that HCQ significantly lowered glycated hemoglobin A1c (3.3%; 95%CI, −3.9 to −2.7, *P* = 0.001) when added to insulin therapy, possibly by improving insulin secretion and peripheral sensitivity [118].

Recently, the use of HCQ has been associated with lower concentrations of serum glucose (85.9 versus 89.3 mg/dl, *P* = 0.04) [119] and a lower incidence of diabetes mellitus in SLE patients, in a dose-dependent manner (HR 0.26; 95%CI 0.18–0.37; *P* < 0.001) [120].

### 8.3. Effects on Thrombosis

HCQ has a protective effect against thrombosis both in SLE patients with and without antiphospholipid antibodies [86]. Such an effect seems mediated by reduced platelet aggregation and protection of the annexin A5 anticoagulant shield from disruption by aPL antibodies [121].

## 9. Discussion

There is good evidence from prospective studies of an increased CV risk in SLE patients [4–7]. Accelerated atherosclerosis, in the presence of traditional risk factors, may explain at least in part this enhanced risk. However, SLE-related factors, as endothelial dysfunction and inflammation, autoantibodies, damage accrual, and disease activity are equally or even more important [10–14]. Such a complex interplay of pathogenetic mechanisms presents clinical challenges, particularly because of the lack of data on the effects of the modification of traditional and SLE-specific CVD risk factors. Presently, in order to lower the CV risk in SLE, the main objectives should be treating the disease targeting remission or low disease activity [122] and sparing corticosteroids when possible, whilst monitoring traditional CVD risk factors at least once a year [123].

HCQ should be an essential part of SLE treatment strategy and should be started as soon as the diagnosis has been made and maintained for an indefinite period if toxicity does not occur [81]. Although for a long time it has been considered a minor component in the management of SLE, in fact, increasing evidence demonstrates that HCQ has a broad spectrum of beneficial effects on disease activity, prevention of damage accrual, and mortality [124]. Furthermore, HCQ is thought to protect against accelerated atherosclerosis by means of several mechanisms of action targeting both SLE-related and traditional CV risk factors.

One of the main limitations to be considered, when interpreting the available data, is the lack of a direct demonstration of the cause-effect relationship between HCQ treatment and atheroprotection from randomized controlled trials. On the other hand, given the many evidences of beneficial effects on HCQ in SLE patients, a placebo-controlled trial would be probably not ethically sustainable. Studies addressing the potential effect of HCQ on CV risk in patients with no existing rheumatic disease with a very high risk of a recurrent CV event, such as the OXI trial (NCT02648464), may shed some light on mechanistic insights regarding the cardioprotective effect of HCQ [125].

In conclusion, despite the lack of randomized controlled trials, the available evidence strongly suggests that HCQ exerts beneficial effects against atherosclerosis and CVD in SLE patients.

## Figures and Tables

**Table 1:** Possible protective effects of HCQ on the interplay between atherosclerosis and SLE pathogenesis.

Features of SLE pathogenesis	HCQ	Features of atherosclerosis pathogenesis
Imbalance between endothelial damage and repair mechanisms	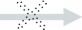	Endothelial dysfunction
Increased oxidative stress	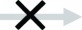	Endothelial damage and impaired vasodilatation
Increased macrophage activation	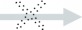	Monocyte recruitment and activation in atherosclerotic plaques
Hyperactive T-cell with increased survival	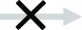	T-cell recruitment and activation in atherosclerotic plaques
Dysregulation of TLR2 and TLR4 activation; activation of TLR7 and TLR9 by anti-DNA	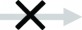	Overexpression and activation of TLRs (especially TLR2/TLR4)
Increased levels of IFN*α*	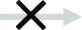	Increased activation of macrophages and foam cells in the atherosclerotic plaques
Increased levels of TNF-*α*, IL-17, IL-6	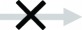	Increased macrophage activation, adhesion molecule expression, chemotaxis, and inhibition of SMC proliferation
Increased levels of IFN-*γ*	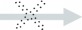	Increased expression of adhesion molecule expression and inhibition of SMC proliferation and collagen production
Increased prevalence of anti-ApoA-1 antibodies and proinflammatory HDL	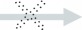	Decreased antiatherosclerosis HDL function

The arrows represent the interplay between SLE and atherogenesis. The crosses represent the proved (black) or potential (blank) action of HCQ in inhibiting the proatherogenic effect of SLE.
